# A Huge Pelvic-Abdominal Malignant GIST Tumour in a Patient with Neurofibromatosis Type 1: Case Report and Literature Review

**DOI:** 10.1155/2020/6590307

**Published:** 2020-01-04

**Authors:** Islam Omar, Hani Alsaati, Ejaz Waris

**Affiliations:** ^1^Furness General Hospital, UHMBT, NHS, UK; ^2^Oncology Center, King Hamad University Hospital, Bahrain

## Abstract

Gastrointestinal stromal tumours are rare tumours of the gastrointestinal tract (GIT) accounting for 0.1%–3% of all gastrointestinal tumours. The most common location is the stomach (55%) followed by the small bowel (31.8%), colon (6%), other various locations (5.5%), and the oesophagus (0.7%). They may also occur in extraintestinal locations. The signs and symptoms of GIST depend on the tumour's location and size. Gastrointestinal bleeding is one of the most common symptoms. Other signs and symptoms include abdominal discomfort, pain or distention; intestinal obstruction, and weight loss. The association between the development of GISTs and neurofibromatosis 1 (NF1) has been established. NF1-associated GISTs tend to have a distinct phenotype, and the absence of KIT/PDGRF*α* mutations in turn has implications on further management when they do not respond well to imatinib treatment. Here, we present one of the largest GISTs reported in the literature with a total volume of 25.3 × 20 × 14 cm + 27.9 × 23 × 8 cm and an overall weight of 7.3 kg, which developed in a 43-year-old female patient with NF1 and was resected on an emergency basis due to the rapid deterioration and development of abdominal compartment syndrome. Pathology assessment showed a malignant GIST composed of spindle cells with elongated nuclei with necrosis, marked pleomorphism and numerous giant cell. The mitotic count was >15/50 HPF, Ki 67 was 80%, and the lymphovascular invasion was clear. Immunohistochemistry investigations showed that Vimentin, CD117, and DOG1 were positive, while BCL-2 and CD99 were focal positives. Pan-CK, S-100, CD34, Desmin, SMA, and HMB-45 were negatives.

## 1. Introduction

Gastrointestinal stromal tumours are rare tumours of the GIT (see comment above) accounting for 0.1%–3% of all gastrointestinal tumours [1, 2]. The most common location is gastric (55%) followed by the small bowel (31.8%), colon (6%), other various locations (5.5%), and the oesophagus (0.7%) [3]. They may also occur in the extraintestinal locations like the mesentery and omentum and in exceptionally rare sites like the gallbladder, urinary bladder, and prostate [4].

The tumour shows no strong sex predilection and usually occurs in adults [5], with a mean reported age of 50.6 years [6]. GIST is extremely uncommon in children and adolescents [7]. Some instances of GISTs show a pattern of familial incidence suggestive of genetic predisposition, whereas others are associated with neurofibromatosis and Carney's triad [5].

GISTs are believed to arise from the interstitial cells of Cajal or related stem cells [8]. In most cases of GISTs, there is a mutation of C-KIT oncogene, which is responsible for the formation of a protein called KIT. Accordingly, KIT (CD117) positivity is observed in 95% of cases [2]. Platelet-derived growth factor receptor alpha (PDGFRA*α*) mutations play a role in GIST pathogenesis [9].

Benign GISTs outnumber the malignant ones by a margin of 10 : 1. Clinical presentation depends on the site and size of the tumour and may include abdominal pain, gastrointestinal bleeding from ulceration of the overlying mucosa, or signs of obstruction. However, small tumours may be asymptomatic. The major clinical findings are upper abdominal ulcer-like pain, dyspepsia, iron-deficiency anaemia, gastrointestinal bleeding, nausea, vomiting, palpable abdominal mass, and weight loss [10, 11]. Also, GISTs with an intra-abdominal abscess have been reported [12, 13].

Many studies confirmed the association between the development of GISTs and neurofibromatosis 1 (NF1) [14–17]. GISTs associated with NF1 seem however to have a distinct phenotype and the absence of KIT/PDGRF*α* mutations which has, in turn, an implication on further management where they do not respond well to Imatinib treatment [18–20].

Here, we present a case of a huge pelvic-abdominal GIST in a known patient of NF1 who showed rapid progression over a short time mounting to abdominal compartment syndrome. Consequently, she underwent emergency surgical excision.

## 2. Case Presentation

**Figure 1:**
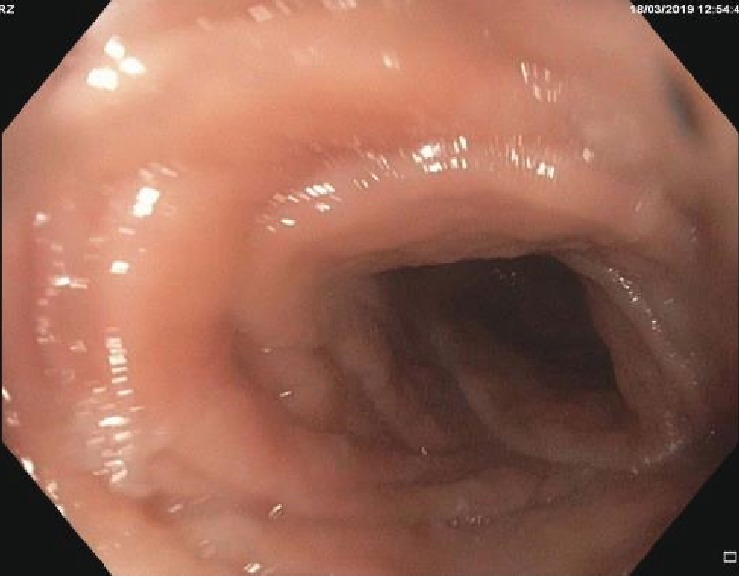
External compression on the sigmoid and descending colon with neither obstruction nor intraluminal lesion.

A 42-year-old female is a known case of familial NF1. She and her identical twin in addition to her only brother are all affected by NF1. She has no family history of malignancy, has not had previous surgery, and has no other comorbidities. Initially, she presented with fatigue, cough, and shortness of breath. She sought medical advice, and a low haemoglobin of 4 g/dl was discovered. She was resuscitated and received a blood transfusion. She had a history of constipation and change of stool calibre and melena. Clinical examination showed an abdominal mass which was confirmed by the US ultrasound later. An urgent OGD and a colonoscopy were done. OGD revealed normal upper GI study while the colonoscopy showed external compression on the sigmoid and descending colon with neither obstruction nor intraluminal lesion (Figure 1).

**Figure 2:**
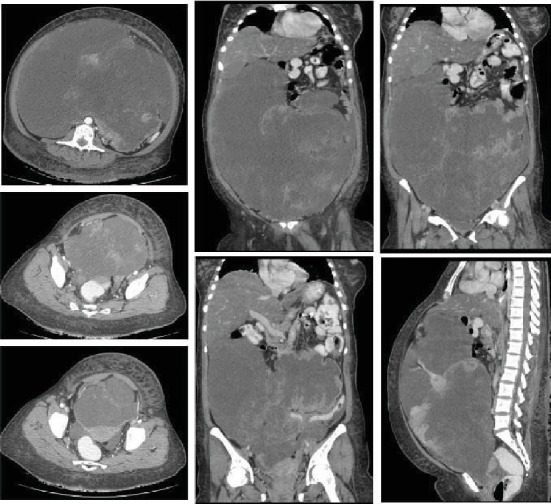
A huge cystic mass occupying almost the whole pelvic and abdominal cavity. The mass is lobulated with enhancing the peripheral soft tissue components and septations, displacing all bowel posteriorly.

After stabilisation, the patient was then referred to our tertiary oncology centre for further evaluation. A CT scan (Figure 2) was done which revealed a huge cystic mass occupying almost the whole pelvic and abdominal cavity, which was lobulated with enhancing peripheral soft tissue components and septations. Moreover, a mild free-flowing peritoneal fluid was noted with diffuse subcutaneous tissue oedema. Of note, there were no signs of metastasis to the liver, lung, or the bones, and the scanned parts of the bowel loops showed no abnormalities.

**Figure 3:**
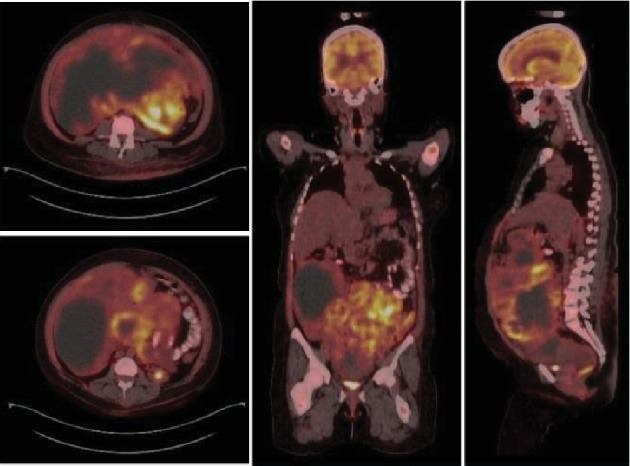
Whole-body PET CT showing multiple amalgamated masses exhibiting peripheral patchy FDG activity with low-grade FDG avid areas of peritoneal thickening/fat stranding suggesting peritoneal involvement. Low-grade FDG avid small prehepatic node measures 8 mm with diffusely activated bone marrow.

Tumour markers were done and showed AFP (2.8 *μ*g/L), CA 125 (154.7 U/ml), CA 15-3 (10.5 U/ml), CEA (0.3 *μ*g/L), CA 19-9 (5.4 U/ml), and B-HCG (0.9 mIU/ml). For staging, a whole-body PET CT scan (Figure 3) was done and showed multiple amalgamated, well-defined, oval-to-round soft tissue masses extending from right subhepatic and lumbar regions just behind the anterolateral aspects of the anterior abdominal wall down to the pelvis, compressing the bowel loops. These masses exhibited heterogeneous fluid and soft tissue densities as well as peripheral patchy FDG activity.

They were associated with low-grade FDG, avid areas of peritoneal thickening, and fat stranding with pelvic ascites, suggesting peritoneal involvement. Also, a low-grade FDG avid small prehepatic node was detected which measured 8 mm in diameter. Additionally, features of a diffusely activated bone marrow were noted, likely proliferative in response to anaemia.

Given the previous scans, the origin of the mass was not clear and was thought to be of ovarian origin. The patient was being optimised and prepared for surgery. At that time, melena was stopped, but the patient continued to complain of shortness of breath, constipation, abdominal pain, and distention.

In an attempt to fully optimize the patient for definitive surgery, all the efforts were carried out to ameliorate the abdominal distention and the mass effects through NGT, urinary catheterization, and strict fluid balance management. Unfortunately, the mass effect was out of control due to the huge size of the tumour and the patient started to experience tachycardia and tachypnea. Abdominal pressure measurement showed a pressure approaching 26 mmHg, a value that met a grade IV ACS [21].

**Figure 4:**
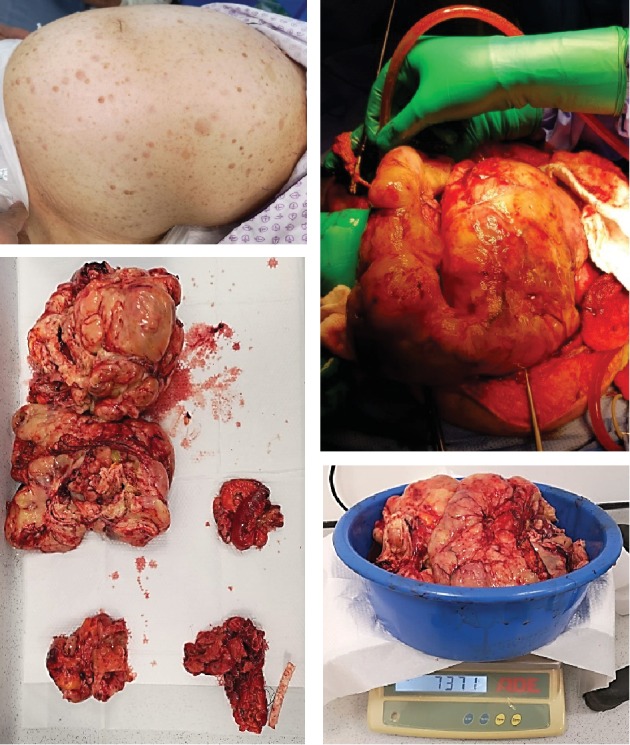
Intraoperative findings showing a huge lobulated mass, occupying most of the pelvic and abdominal cavities with the involvement of the sigmoid colon and small bowel. The weight of the resected mass exceeded 7.3 kg.

At that stage, the decision was taken to do emergency surgery to avoid the expected consequences of ACS [22]. Exploratory laparotomy was performed after bilateral ureteral stenting. A huge intra-abdominal mucinous mass was found (Figure 4) coming from the pelvis with no clear origin. There was involvement of the peritoneum, small bowel, sigmoid colon, and upper rectum. Moreover, multiple areas of the small bowel were involved, around 80 cm from the ileocaecal junction. Therefore, excision of the mass was performed with bilateral salpingo-oophorectomy. Since the sigmoid colon was found adherent to the tumour, resection of the sigmoid colon and colostomy creation were done. Moreover, the involved segments of the small bowel were resected and primarily anastomosed. The grossly involved peritoneum was resected.

**Figure 5:**
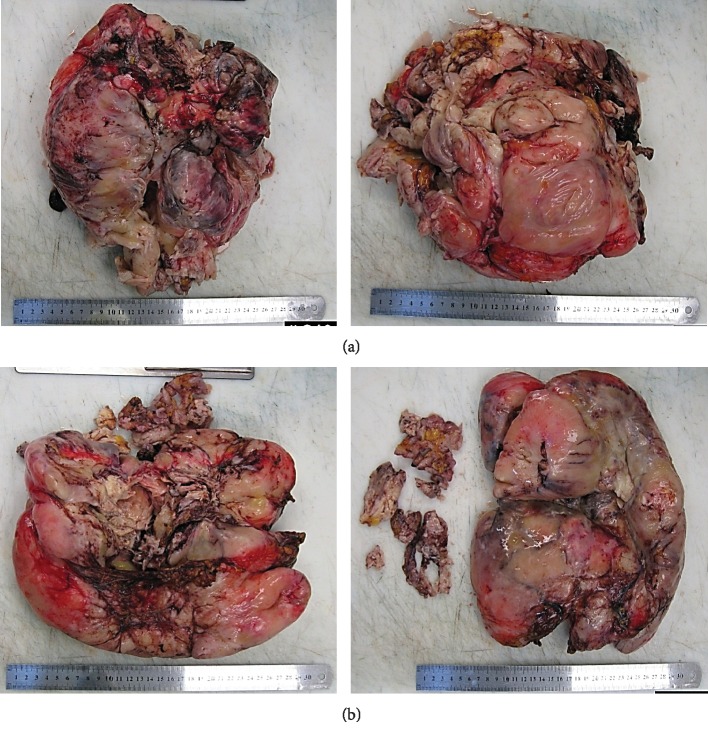
(a) Gross pathology of the first mass showing a huge mass with dimensions of 25.3 × 20 × 14 cm which was greyish white in color with nodular surface and partially capsulated. There were multiple foci of hemorrhage, congestion, and necrosis. (b) Gross pathology of the second mass measuring 27.9 × 23 × 8 cm. Its external surface was nodular, partially capsulated, and showed foci of congestion, haemorrhage, and necrosis.

On gross pathological examination, the overall weight of the specimen was 7.3 kg. The first specimen (Figure 5(a)) was a huge mass with dimensions of 25.3 × 20 × 14 cm. The mass was firm, greyish white with nodular surface, and partially capsulated. There were multiple foci of haemorrhage, congestion, and necrosis. The cut surface of the mass showed a variegated appearance with focal greyish white areas. There were also focal areas of mucinous cystic degeneration, haemorrhage, and necrosis. A small bowel segment 8.5 cm in length by 3.2 cm in circumference was found adherent to the mass. On opening the bowel segment, its mucosa was unremarkable. However, the serosal surface adhered to the mass. The second specimen (Figure 5(b)) came with dimensions of 27.9 × 23 × 8 cm. The external surface of the mass was nodular, partially capsulated, and showed foci of congestion, haemorrhage, and necrosis. Along with that, an exudative membrane was found on the surface. The cut surface was glistening, shiny, and having a variegated appearance with focal areas of haemorrhage and necrosis in addition to marked cystic degeneration.

**Figure 6:**
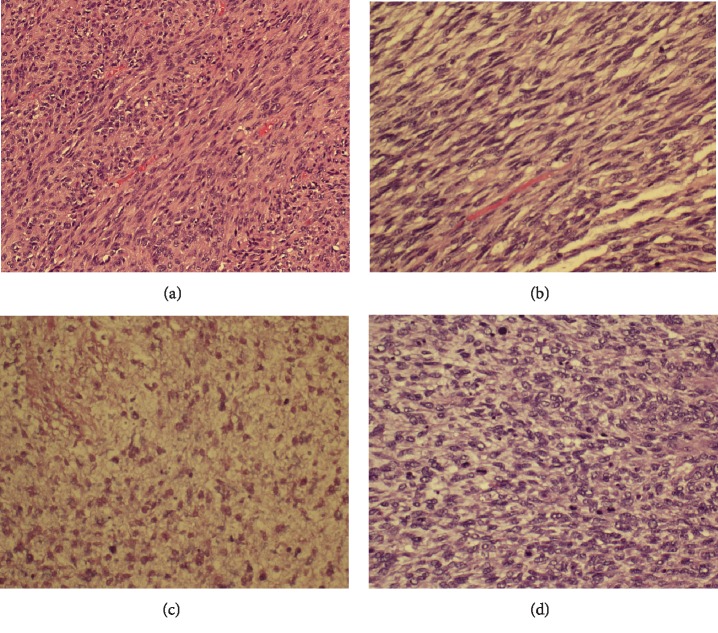
Histopathology of the tumor. (a, b) The tumor composed of spindle cells with elongation. (c) Areas with necrosis. (d) High mitotic figures.

Histopathology examination (Figure 6) of the two huge masses showed features of a malignant neoplasm. It was composed of spindle cells with elongated nuclei arranged in fascicles. Moreover, there was marked pleomorphism and numerous tumour giant cells. The mitotic count was >15/50 HPF. Additionally, there were poorly differentiated areas with 20% necrosis. The tumour was of the high-grade type.

**Figure 7:**
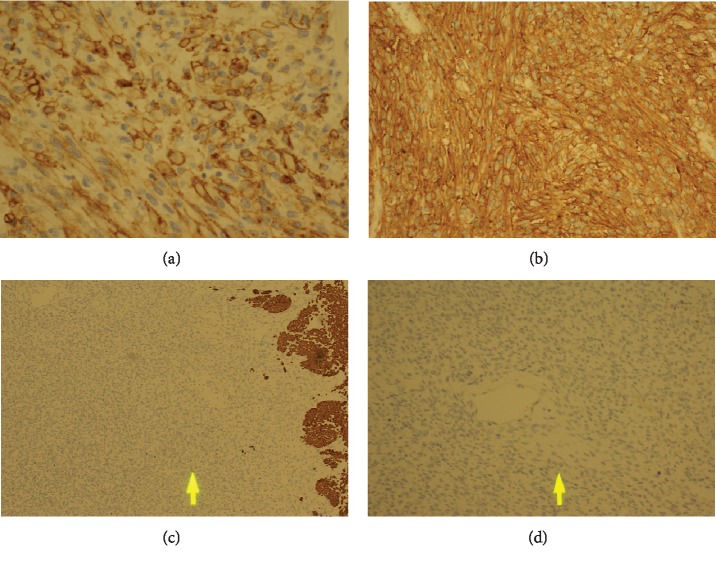
Immunohistochemistry staining. (a) Positive CD117. (b) Positive DOG1. (c) Desmin negative. (d) S100 negative.

Risk assessment according to Fletcher et al.'s criteria [23] showed that the tumour was of high risk. Margins of the resected small and large bowel loops were free from tumour invasion. However, evidence of lymphovascular invasion was clear. Immunohistochemistry investigations (Figure 7) showed that Vimentin, CD117, and DOG1 were positives, BCL-2 and CD99 were focal positives, Pan-CK, S-100, CD34, Desmin, SMA, and HMB-45 were negative, and Ki-67 was 80%.

Two segments of the small bowel were resected weighing 675 g. The first segment measured 26 cm in length by 4.8 cm in circumference. A polypoid, soft-to-firm, and tan-to-grey in colour mass was found adherent to its serosal surface measuring 8 × 5.2 × 1.3 cm. It was 10.1 cm away from the closest margin, and 17.7 cm away from the distant margin. On opening the small bowel segment, the entire length of mucosa was gangrenous with an area of perforation identified, measuring 7.6 mm, it was 8.5 cm away from the closest margin.

The second segment of the small bowel measuring 27.9 cm in length and a 5.3 cm circumference, with a polypoid grey soft-to-firm mass, was found adherent to the serosal surface, measuring 36.5 × 6.7 × 3.4 cm. The mass was 9.5 cm away from the closest margin and 9.6 cm away from the distant margin. Grossly, it did not appear to involve the mucosa. On opening the lumen, the mucosal surface, at the level of mass attached to the serosa, was flattened and the remaining mucosa showed an area of stricture measuring 2.5 × 1 cm, 4.3 cm away from the closest margin with no perforation. Histopathology examination of the bowel loops showed thinning out of the wall with tumour infiltration into the mesenteric fat, reaching up to the muscular layer but the resection margins were negative.

One segment of the sigmoid colon was included and measured 27.9 cm in length by 8 cm in circumference. The specimen was received with an attached mesentery and weighed 299 g. A polypoid grey tan firm mass was found adherent to the mesenteric fat, measuring 10.5 × 3.6 × 2.1 cm, and it was 2.1 cm away from the closest margin. Upon opening the lumen, the mucosal surface along the entire segment was normal with no strictures, perforation, or diverticulosis. Histopathology examination of the sigmoid colonic wall showed tumour infiltration into the mesenteric fat reaching up to the muscular layer with four reactive lymph nodes. The resection margins were clear.

Another specimen was retrieved and included two pieces of the peritoneum. The first from the right side measured 13 × 5.3 × 2.2 cm along with a nodule measuring 2.8 × 2 × 1.5 cm and both weighed 47 g. The second piece came from the left peritoneal layer which measured 18 × 5.5 × 0.8 cm and weighed 25 g. The right peritoneum specimen was positive for tumour infiltration where sections revealed tumour infiltration of the fibrofatty tissue. The left peritoneal layer showed small ectopic adrenal tissue and was positive for tumour infiltration.

Specimens of the ovaries and fallopian tubes revealed a left ovary measuring 3.5 × 2.4 × 1.2 cm with the left fallopian tube measuring 5 × 1.2. The overall weight of the left ovary and fallopian tube was 12 g. The right ovary and the attached fallopian tube with surrounding fibrofatty tissue weighed 9 g. The right ovary measured 3.2 × 1.8 × 1.4 cm. The attached right fallopian tube measured 4.2 × 1.2 cm. The attached fibrofatty tissue showed marked areas of congestion. Histopathology examination of the left ovarian mass revealed a normal ovary and fallopian tube with tumour infiltration into the paratubal tissue. The right ovarian mass was free of invasion.

The conclusion of the pathology assessment came as high-risk GIST (pT4, pN0, pMx) with tumour invasion into the left paratubal tissue, small and large bowel, in addition to the right and left peritoneal tissues. The patient had an uneventful postoperative course and was discharged home. Her case was discussed in the national tumour board—MDT, which recommended starting Imatinib therapy and genetic analysis. Unfortunately, genetic analysis was not available in any local centre. Every effort was made to send the tissue blocks abroad for genetic analysis; however, logistical obstacles aborted these endeavours.

Although the patient received the first dose of Imatinib, her condition started to deteriorate over the next 2 months after surgery with persistent vomiting and gradual abdominal distention. However, her stoma was functioning and a barium follow-through study confirmed the patency of her bowel. Because of the multiple admissions due to persistent vomiting and abdominal distention, a CT scan was done which confirmed an aggressive recurrence. Because of the severe distention and again abdominal compartment syndrome, the patient was taken to the theatre where an aggressive inoperable tumour was encountered. Laparostomy was performed. The abdomen was left open and covered with a Bogota bag. A few days after the second surgery, the patient passed away.

## 3. Discussion

The signs and symptoms of GIST depend on the tumour's location and size, with highly malignant GISTs typically being large and symptomatic at the time of diagnosis. Gastrointestinal bleeding is one of the most common symptoms. Other signs and symptoms include abdominal discomfort, pain or distention, intestinal obstruction, and weight loss [24].

In this case, there was a typical presentation with anaemia, melena, and gradual abdominal distention. Although there was no complete obstruction, the patient gave a history of change of stool calibre and constipation, manifestations that go with the external compression exerted by the huge tumour on the bowel.

GISTs arise in the muscularis propria layer of the stomach or intestinal wall. Small GISTs form intramural or serosal nodules and, as they grow and expand, may develop intraluminal, intramural, and extraluminal components to varying degrees. The extraluminal component of a malignant GIST may be so large that it is difficult to determine the site of origin [24, 25].

On the radiological basis, the tumour in our patient was first thought to have originated from the ovaries due to its pelvic-abdominal position and close proximity to the gonads. However, on histopathology assessment, it was proved to invade the left paratubal tissue planes only. Given its huge size and invasion of both the sigmoid colon as well as the small bowels, the origin is not clear; however, the invasion to the small bowel was more aggressive with perforation and stricture formation suggesting a small bowel origin.

Given the weight and volume of our tumour (overall weight of 7.3 kg—25.3 × 20 × 14 cm for the first mass and 27.9 × 23 × 8 cm for the second mass), it is one of the largest GISTs reported in the literature. Koyuncuer et al. [26] reported a huge GIST originating from the stomach in a 43-year-old male, which was 6.109 kg in weight and measured 39 × 27 × 14 cm. Also, Cappellani et al. [27] reported a gastric GIST in a 67-year-old male, weighing 8.5 kg and measuring 37 × 24 × 13 cm. Moreover, Cruz et al. described a gastric GIST in a 37-year-old male, which was 32 × 25 × 21 in size and 3.75 kg in weight. Accordingly, to the best of our knowledge, our patient had the largest reported GIST in the literature with a volume of 25.3 × 20 × 14 + 27.9 × 23 × 8 cm and the second heaviest tumour weighing 7.3 kg.

GISTs are potentially aggressive forms of cancer that may develop anywhere in the GI tract [28]. They most frequently metastasize within the abdominal cavity, especially the liver and peritoneum. Bone and lung metastases are far less common [28, 29]. Very rare metastases to the skeletal muscles, adrenal gland, brain, testicles, and heart have also been reported [30–33]. Sizes larger than 10 cm cystic changes, high cellularity, mitotic figures >10/50 HPF, and coagulative necrosis are all more likely to be associated with metastasis [34].

In our patient, the histopathology assessment classified the tumour as a high-risk malignant tumour. Although distant spread to the liver or bones is excluded based on radiological staging, the tumour is proved to be locally aggressive spreading to the peritoneum, bowels, and paratubal tissues. This is expected given the huge nature of the tumour, a mitotic count of >15/50 HPF, and Ki-67of 80%.

Association between GIST and NF1 is established since an old autopsy study had documented a GIST in one-third of the NF1 patients [35]. NF1-associated GISTs are typically reported as multiple, generally low-grade tumours that affect the jejunum, ileum, duodenum, and stomach [15, 36]. Salvi PF et al. [19] in a systematic review of 252 patients with NF1-associated GISTs reported that patients affected by NF1-associated GISTs were younger and tumours were significantly smaller. Moreover, tumours were located mainly in the jejunum and ileum in the NF1 subgroup, whereas the main localization in the sporadic group was the stomach. They also reported a prevalence of low-risk criteria in the NF1 subgroup compared with the sporadic GISTs.

In our case, the patient was young (43 years old), with the tumour most probably originating from the small bowel—criteria that go with the previous literature. However, the tumour was huge and of the high-risk category, which were both striking features.

The genetic basis for the association between NF1 and GISTs is being elucidated. The genetic defect of NF1 disease is a mutation in the NF1 gene which encodes neurofibromin, a tumour suppressor protein that regulates the cellular proliferation via inactivation of RAS through GTPase activity. RAS is known to stimulate signal transduction through the MAP kinase pathway when activated. The resultant loss of the function of neurofibromin predisposes to the development of benign and malignant tumours [37–39]. NF1-related GI disease has been described to occur in three principal forms: neurogenic tumours, stromal tumours, and neuroendocrine tumours [40–42]. The most common of these GI manifestations in NF1 is GIST with one study indicating an incidence of 7% in the NF1 population and another reporting a 150-fold increased risk as compared to the general population [35, 38].

GISTs in adults are typically sporadically occurring and are associated with somatic mutations in the KIT or PDGFR*α* gene. However, 10%–15% of GISTs lack KIT or PDGFR*α* mutations. These GISTs can be associated with NF1 or can represent succinate dehydrogenase (SDH)-deficient GISTs that include GIST in the Carney triad and the Carney-Stratakis syndrome [43, 44].

GISTs associated with Carney Triad, Carney Stratakis syndrome, along with young and paediatric GISTs have been documented to have a loss of succinate dehydrogenase subunit B (SDHB) expression [20, 45, 46].

Based on the SDHB expression, it has been recently proposed that GISTs could be differentiated into two characteristic subgroups: type 1 SDHB-positive and type 2 SDHB-negative [45]. SDHB-positive GISTs usually occur in adults with no predilection of the tumour's locations, show homogeneous male-to-female ratio, present KIT or PDGFR*α* mutations, and, generally, may benefit from the Imatinib treatment. SDHB-negative GISTs occur usually in paediatric and young female patients, locate almost exclusively in the stomach and present an epithelioid morphology. These tumours are usually c-kit and PDGFR*α* wild-type and do not respond to the molecular treatment with Imatinib [47, 48].

Even though NF1-associated GISTs have been documented to be type 1 SDHB-positive tumours [20], they could be differentiated by several features including the predilection of localization to the jejunum and small intestines, common tumour multiplicity, the lack of GIST-specific mutations (kit and PDGFR*α* wild type); and they are KIT-positive with hyperplasia of ICCs. Moreover, and alike the SDHB type 2 tumours, they do not respond well to Imatinib treatment [15, 20, 49–51].

Given the above, the therapeutic challenges for advanced NF1-associated GISTs are evident with expected resistance to Imatinib therapy. However, Sunitinib is the second-line tyrosine kinase inhibitor and has shown activity with clinical benefit in 56% of wild-type patients and can be used for the patients who develop resistance to Imatinib as well [52].

## 4. Conclusion

NF1-related GISTs represent a unique entity of GISTs relevant to their different molecular basis and presentation. When advanced and unresectable, they pose a great challenge due to resistance to Imatinib therapy. Here, we present one of the largest GISTs reported in the currently available literature with an overall weight of 7.3 kg and volume of 25.3 × 20 × 14 + 27.9 × 23 × 8 cm, which was resected on an emergency basis due to the rapid deterioration and development of abdominal compartment syndrome.
